# Effect of flupirtine on the growth and viability of U373 malignant glioma cells

**DOI:** 10.7497/j.issn.2095-3941.2013.03.004

**Published:** 2013-09

**Authors:** Elango Panchanathan, Gnanasambandan Ramanathan, Bhaskar Venkata Kameswara Subrahmanya Lakkakula

**Affiliations:** 1 Department of Pharmacology, Sri Ramachandra University, Chennai 600118, India; 2 Department of Biomedical Sciences, Sri Ramachandra University, Chennai 600118, India

**Keywords:** Flupirtine, N-Methylaspartate, analgesics, glioma

## Abstract

**Objective:**

Flupirtine is a non-opioid analgesic without antipyretic or antiphlogistic properties but with favorable tolerability in humans. This analgesic also exhibits neuroprotective activities. Furthermore, flupirtine antagonizes glutamate- and NMDA-induced intracellular levels of Ca^2+^ and counteracts the effects of focal cerebral ischemia. Although flupirtine has been used to relieve pain caused by different diseases and clinical procedures, information on the safety and efficacy of flupirtine is limited. The present study was conducted to investigate the neuroprotective effects of flupirtine on U373 malignant glioma (MG) cell lines.

**Methods:**

Cell viability and cell cycle analysis was performed by MTT assay and flow cytometry, respectively.

**Results:**

Variations in the growth of U373 MG cells in 5 mM *N*-methyl-D-aspartate (NMDA), 1 mM flupirtine, and combined treatment indicated the antagonistic effects of NMDA and flupirtine on MG cell lines. The variation in the percentage of gated cell population in different cell cycle phases showed significant variations after 48 h of treatment.

**Conclusion:**

Flupirtine has neuroprotective effect of on U373 MG cells, which limits its use in the pain management of brain tumors. This property warrants further studies using animal models and large-scale clinical trials.

## Introduction

The treatment of pain caused by disease or surgery poses a great challenge for clinicians. In general, non-steroidal anti-inflammatory drugs and opioids are used to manage pain. For instance, flupirtine is a triaminopyrimidine derivative that mainly functions as a non-opioid analgesic and has been applied effectively in clinical practice since 1984. Flupirtine maleate is water soluble and undergoes rapid gastric absorption in animals^1^ and humans^2^. Flupirtine, administered either orally or rectally, also undergoes biotransformation in the liver and is well tolerated by the body. Furthermore, flupirtine is a relatively safe substance that can be prescribed for children aged less than six years. Studies have also extensively investigated the pharmacological and therapeutic properties of flupirtine against pain at a clinically relevant dosage range; the results showed potent cytoprotective and neuroprotective activities as well as anticonvulsant and myorelaxant effects. Exhibiting muscle relaxant properties, flupirtine effectively relieves low back pain and other orthopedic conditions^3^.

Flupirtine rarely elicits side effects; when manifested, these side effects include fatigue, drowsiness, dizziness, headache, nausea, and vomiting. This analgesic has been used for the treatment of various neurological disorders involving neuronal overexcitability, such as epilepsy and neuropathic pain^4^, and human prion diseases^5^. In addition, the pharmacodynamic effects of flupirtine are related to selective neuronal potassium channel opening activity and *N*-methyl-D-aspartate (NMDA) receptor antagonist property^6^. Flupirtine also activates the descending noradrenergic pain-modulating pathways^7^, such as GABA (A) receptors and Kv7 channels^8^. Studies using animal models have further revealed that flupirtine completely protects neurons from apoptotic cell death particularly in rats^9^. Flupirtine also antagonizes glutamate- and NMDA-induced intracellular levels of Ca^2+^ and counteracts the effects of focal cerebral ischemia in mice^10^. As such, flupirtine has been used to manage pain caused by different diseases and clinical procedures for several years in Europe; however, information on the safety and efficacy of flupirtine is minimal. This analgesic has not been approved by US FDA for effective use in clinical practice. Hence, the present study was conducted to investigate the neuroprotective effects of flupirtine on malignant glioma (U373 MG) cell lines.

## Materials and methods

### Cell culture and maintenance

U373 MG cell lines were obtained from the National Centre for Cell Sciences (NCCS), Pune, India. These cell lines were cultured in Dulbecco’s Modified Eagle Medium supplemented with 100 units/mL penicillin, 100 g/mL streptomycin, and 10% heat-inactivated fetal bovine serum. The cells were maintained at 37 °C in a humidified atmosphere with 5% CO_2_ and 95% air.

### Analysis of cell viability

Cell viability was assessed using 3-(4,5-dimethyl-2-thiazolyl)-2,5-diphenyl-2H tetrazolium bromide (MTT) colorimetric assay (Sigma-Aldrich). U373 MG cells were seeded into a 96-well flat bottom plate at a density of 1×10^3^ cells per well. The cells were treated with 5 mM NMDA, 1 mM flupirtine, and combined 5 mM NMDA and 1 mM flupirtine for 24 and 48 h. Control cell cultures were prepared without treatment of flupirtine and NMDA. After incubation, the medium was removed; MTT reagent (0.5 mg/mL final concentration) was then added to each well and incubated for 4 h. The control cells were treated with dimethyl sulfoxide. Insoluble formazan crystals were solubilized by adding 100 µL of 100% acidic alcohol solution. The plates were read at 570 nm by using an automatic microtiter plate reader (Bio-Rad). Growth inhibition of 50% (GI_50_) was determined by comparing the values of treated and control samples.

### Flow cytometric analysis

U373 MG cultured cell lines were seeded into six-well plates at a density of 2×10^5^ cells to 3×10^5^ cells per well. After 24 h of incubation, these cells were exposed to NMDA, flupirtine, and combined treatment for 24 and 48 h under the same conditions used to detect cell viability. The percentage of apoptotic cells in the total cell population (adhering+detached cells) was evaluated as previously described^11^. In brief, the cells were collected, washed, and centrifuged at 453 *g* for 10 min. The cell pellet was resuspended in 1 mL of hypotonic fluorochrome solution (50 µg/mL propidium iodide in 0.1% sodium citrate plus 0.1% Triton X-100). After 30 min, the cells were analyzed using a flow cytometer (FACS Calibur, Becton Dickinson, USA) equipped with an air cooled argon laser at 15 mW and 488 nm in a standard filter setup. For each sample, 10,000 events were acquired and the percentage of each cell cycle phase was determined using CellQuest Pro software (Becton Dickinson).

### Statistical analysis

The results were summarized as mean±standard deviations. Using SPSS, we performed *F* test and Levene’s test to determine whether or not variances are different among the three groups. Differences were considered statistically significant at *P*<0.05.

## Results

### MTT assay

**Figure 1:**
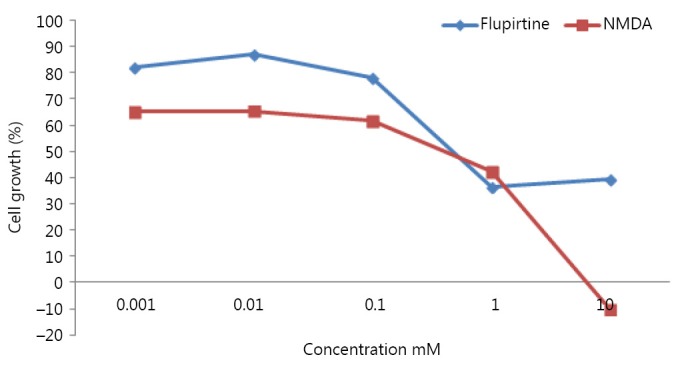
Cell viability of U373 malignant glioma after treatment with flupirtine and NMDA.

The initial experiments aimed to investigate whether or not flupirtine, NMDA, and combined treatment can affect the viability of U373 MG cell lines. The concentrations ranged from 0.001 to 10 mM for 24 h. GI_50_ of flupirtine and NMDA were 0.47 and 0.4, respectively. The growth of U373 MG cells was significantly reduced at high doses (1 and 10 mM) of flupirtine compared with low doses (0.001 to 0.1 mM) and control dose (*P*<0.001; **Figure 1**). NMDA elicited adverse damaging effects on the growth of U373 MG cells at 10 mM compared with the control treatment (*P*<0.001; **Figure 1**).

### Effect of flupirtine and NMDA on cell cycle

**Figure 2:**
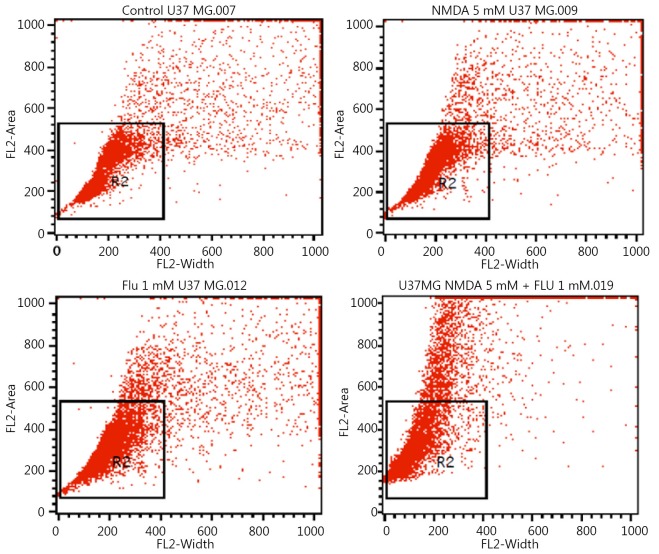
Flow cytometry analysis results of the effect of flupirtine, NMDA, and combined treatment on the cell cycle phases.

**Figure 3:**
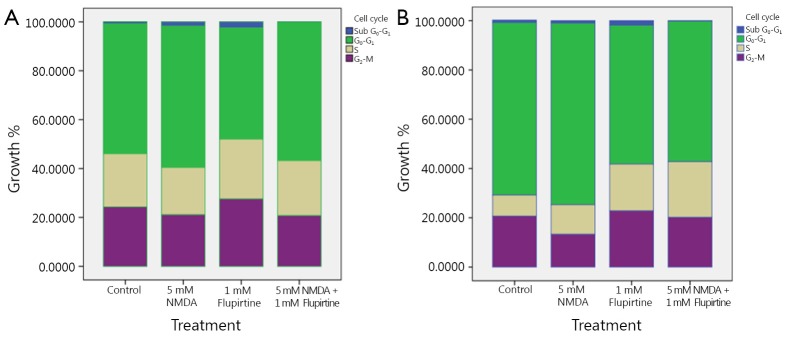
Distribution of gated cell population in different cell cycle phases after treatment with flupirtine, NMDA, and their combination (A. 24 h; B. 48 h).

**Figure 2** shows the gated U373 MG cell population in response to flupirtine, NMDA, and combined treatment. **Figure 3** illustrates the effect of flupirtine, NMDA, and combined treatment on the cell cycle phases of U373 MG cells for 24 and 48 h of incubation. In particular, the cells treated with 5 mM NMDA for 24 h showed a higher percentage of G_0_-G_1_ cell cycle phase than the control cells (**Figure 3**). The flupirtine-treated cells showed lower G_0_-G_1_ cell cycle phase at 1 mM than the control cells. The percentage of gated cell population in different cell cycle phases varied after 24 and 48 h of treatment (**Table 1**). Levene’s test and F-test results showed that the significant variations in cell cycle phases were found only after 48 h but not after 24 h of treatment. The combined NMDA and flupirtine treatment decreased the percentage of cells at G_0_-G_1_ cell cycle phase compared with NMDA alone. The cells treated for 48 h showed the same antagonistic effects of NMDA and flupirtine drugs compared with the control cells and those treated at 24 h. The percentage of cells in G_0_-G_1_ cell cycle phase increased in NMDA-treated cells but decreased in flupirtine-treated cells. The combined treatment reduced the percentage of cells in G_0_-G_1_ cell cycle phase but increased the percentage of cells of other cell cycle phases (**Figure 3**).

**Table 1:** Variation in the percentage of gated cell population in different cell cycle phases after 24 h and 48 h of treatment

Cell cycle stage	24 h treatment	48 h treatment
Control		
Sub G_0_-G_1_	0.91±0.20	1.00±0.00
G_0_-G_1_	53.16±1.84	70.06±2.64
S	21.69±1.05	8.54±1.12
G_2_-M	24.33±0.68	20.65±1.13
5 mM NMDA		
Sub G_0_-G_1_	1.78±0.26	0.96±0.06
G_0_-G_1_	57.84±2.01	73.72±1.72
S	19.29±1.42	12.02±0.90
G_2_-M	21.12±0.70	13.31±1.06
1 mM Flupirtine		
Sub G_0_-G_1_	2.49±0.30	1.82±0.80
G_0_-G_1_	45.48±1.60	56.39±5.60
S	24.47±1.45	18.99±0.96
G_2_-M	27.56±1.08	22.80±1.86
5 mM NMDA+1 mM flupirtine		
Sub G_0_-G_1_	0.35±0.10	0.19±0.05
G_0_-G_1_	56.45±2.20	57.02±3.61
S	22.43±1.15	22.61±1.81
G_2_-M	20.77±0.68	20.19±2.54
Levene’s test of equality of error variances (df=15)		
*F*	1.833	2.997
*P*	0.074	0.004

Values are mean±SD (*n*=3).

## Discussion

The result of our cell viability test using flupirtine, NMDA, and combined treatment revealed the antagonistic effects of NMDA and flupirtine on U373 MG cell lines. In particular, NMDA suppressed the cell cycle at G_0_-G_1_ phase of U373 MG cell lines and further inhibited the cell cycle after flupirtine was added. This result indicated the neuroprotective function of flupirtine.

A previous study compared the analgesic efficacy and safety of flupirtine with those of pentazocine; the results showed that flupirtine is significantly more effective and elicits fewer side effects than pentazocine when these two drugs are used to reduce very severe cancer-induced pain^12^. Hence, flupirtine satisfies the requirements of patients and doctors for effective cancer pain relief ^13^. Further studies have suggested the function of flupirtine in the treatment of neuropathic pain^14^. Different molecular mechanisms may also account for flupirtine-mediated neuroprotection. *In vitro* and *in vivo* studies have suggested that flupirtine antagonizes the neurotoxicity caused by the prion agent PrP^Sc^ and lead acetate [Pb(C_2_H_3_O_2_)_2_^·^3H_2_O] mediated by NMDA receptors^15^. Flupirtine significantly inhibits the neurotoxic effect caused by amyloid *β*-protein segments in Alzheimer’s disease^16^ and other neurological disorders, such as amyotrophic lateral sclerosis^17^. Studies using animal models have also revealed that flupirtine counteracts the effects of retinal and cerebral ischemia^9^^,^^10^^,^^18^. In another study, the long-term flupirtine treatment of chronic pain prevents retinal ganglion cells from degeneration in a non-inflammatory animal model of optic nerve transmission; this result indicated that this drug is a potential candidate and should be further evaluated in terms of its neuroprotective potential^19^.

Flupirtine induces the expression of anti-apoptotically acting protooncogene Bcl-2 in cultured cortical neurons after excitotoxic neuronal cell death^15^. In addition, the anti-oxidative effects of flupirtine have been demonstrated in rat hippocampal slices^20^. In other studies, flupirtine is compared with other analgesics; the results demonstrated that flupertine more effectively reduces pain than pentazocine^13^^,^^21^^,^^22^, tramadol^23^, paracetamol^24^, and aspirin^25^. Furthermore, flupirtine and diclofenac exhibit the same efficacy against orthopedic post-operative pain^26^ and musculoskeletal pain^27^. The combined therapy of flupirtine and morphine increases antinociceptive activity without causing adverse effects^14^. In clinical trials, pain assessment and treatment with flupirtine have revealed significant reduction in pain. Although large-scale clinical trials have rarely been conducted, current studies indicate that flupirtine effectively reduces chronic musculoskeletal pain, migraine, and neuralgias.

Evidence has shown that cancer cells lack apoptotic characteristics. As such, the neuroprotective effect of flupirtine observed in malignant neuronal cells may further aggravate cancer metastasis. Hence, the neuroprotective effect of flupirtine observed in malignant neuronal cells limits the use of this drug in the pain management of brain tumors. Therefore, the beneficial and potentially harmful effects of flupirtine should be well elucidated for accurate therapeutic use in the pain management of brain tumors. This aspect should be further studied using animal models and large-scale clinical trials.
